# Use of rhodizonic acid for rapid detection of root border cell trapping of lead and reversal of trapping with DNase

**DOI:** 10.1002/aps3.1240

**Published:** 2019-04-12

**Authors:** David A. Huskey, Gilberto Curlango‐Rivera, Martha C. Hawes

**Affiliations:** ^1^ Department of Soil, Water and Environmental Sciences University of Arizona 429 Shantz Building, #38 1177 E. Fourth Street, P.O. Box 210038 Tucson Arizona 85721‐0038 USA

**Keywords:** border cells, extracellular DNA (exDNA) trapping, lead (Pb), rhodizonic acid (RA)

## Abstract

**Premise of the Study:**

Lead (Pb) is a contaminant whose removal from soil remains a challenge. In a previous study, border cells released from root tips were found to trap Pb, alter its chemistry, and prevent root uptake. Rhodizonic acid (RA) is a forensic tool used to reveal gunshot residue, and also to detect Pb within plant tissues. Here we report preliminary observations to assess the potential application of RA in exploring the dynamics of Pb accumulation at the root tip surface.

**Methods and Results:**

Corn root tips were immersed in Pb solution, stained with RA, and observed microscopically. Pb trapping by border cells was evident within minutes. The role of extracellular DNA was revealed when addition of nucleases resulted in dispersal of RA‐stained Pb particles.

**Conclusions:**

RA is an efficient tool to monitor Pb–root interactions. Trapping by border cells may control Pb levels and chemistry at the root tip surface. Understanding how plants influence Pb distribution in soil may facilitate its remediation.

*“One can find in various texts the statement that the root‐cap cells of plants die and are sloughed off, and it is probably the general opinion among botanists that the root‐cap cells are either dead when they are sloughed off or that they die soon after…That the root‐cap cells, when sloughed off, are not necessarily dead or short‐lived but may persist for many days, seems to be substantiated by various observations made by the writer with a number of different plants. In view of the increasing attention being devoted to the subject of root excretions, it seems desirable to make record of these incidental observations.”* *(Knudson,* 1919 *)*

Root border cells comprise a population of differentiated cells programmed to separate from the root tip as it penetrates the soil (Hawes, 1990; Hawes et al., 1998, 2012). A species‐specific number of detached living cells are produced by dedicated meristem initials over a 24‐h period (Niemira et al., 1996; Hamamoto et al., 2006; Curlango‐Rivera et al., 2013). The cells remain appressed to the root surface in the absence of free water, but upon contact with water the surrounding root cap mucilage swells immediately, the cell population disperses within minutes, and newly dividing cells can be seen within the root cap meristem within five minutes (Brigham et al., 1998). Previously known as “sloughed root cap cells” (Knudson, 1919) based on the premise that they die and fall off, border cells are now known to function in protection of the growing root tip by a newly recognized immune response first identified in mammalian systems (Brinkmann et al., 2004). Like neutrophils, metabolically active border cells export extracellular DNA (exDNA)–based traps that protect root tips by immobilizing pathogens and thereby preventing infection (Hawes et al., 2012, 2016a; Wen et al., 2017). Genes encoding extracellular DNases (exDNases), which degrade exDNA, facilitate virulence of bacterial and fungal pathogens in both mammalian and plant systems (Buchanan et al., 2006; McCormick et al., 2010; Tran et al., 2016; Park et al., 2019).

Metals have also been implicated as signals that trigger trap formation. In humans, aluminum induces neutrophil exDNA traps (Munks et al., 2010; Stephen et al., 2017). The responses of border cells from cotton, ferns, cereals, and legume plants to metals such as aluminum, copper, lead (Pb), cadmium, mercury, arsenic, and iron have been documented (reviewed in Hawes et al., 2016b). Snap bean border cell mucilage rapidly expands to form extracellular traps in a dosage‐dependent manner in response to aluminum. Aluminum uptake into the root tip is inhibited by the presence of border cells (Brigham et al., 2001). A similar result was observed in cotton and rice border cells in response to copper (Miyasaka and Hawes, 2001; Curlango‐Rivera et al., 2013; Peng et al., 2016). The role of exDNA has not been examined in this defense response in plants. Accumulation of Pb on “root cap slime,” the mucilage layer covering the root cap surface, was observed previously, but the role of border cells was not evaluated (Sobotik et al., 1998). We recently documented that corn and pea border cell extracellular trap dimensions increase within minutes in response to Pb, that significant levels of Pb are removed from suspension by border cells, and that the chemistry of Pb is altered by interaction with border cells (Huskey et al., 2018). When border cells are present on root tips immersed into Pb, the particles accumulate on the surface of the border cells and no root growth inhibition occurs. In contrast, when the border cells are removed by agitation in water before root immersion into Pb, significant growth inhibition is evident within 24 h.

Given the dynamics and magnitude of the trapping response, a tool that facilitates real‐time visualization of the process could help define how Pb is distributed in and on plant tissues within minutes of exposure. Rhodizonic acid (RA) was developed as a tool to detect the presence of Pb in plant tissue (Glater and Hernandez, 1972). Upon contact, a scarlet precipitate forms with the color intensity proportional to the amount of Pb. RA is used to detect gunshot residue (Di Maio, 1998), as well as to analyze the deposition of Pb in plant tissues (Inoue et al., 2013; Tupan and Azrianingsih, 2016). If exDNA plays a role in Pb trapping, then treatment with DNA‐degrading enzymes would be predicted to result in reversal of trapping that can be visualized by a Pb‐specific stain. In the current study, RA was used to document the dynamics of border cell trapping of Pb, and to illustrate reversal of trapping in response to exDNA degradation by DNase I, DNase II, and S1 nuclease.

## METHODS AND RESULTS

### Plant material

Golden Bantam corn seeds (Victory Seed Company, Molalla, Oregon, USA) were surface sterilized for 5 min in a 95% ethanol solution, followed by 10 min in 4% sodium hypochlorite. Surface‐sterilized seeds were then rinsed six times with sterile deionized water (sdH_2_O) 17.3 MΩ/cm e‐Pure (Barnstead/Thermolyne, Dubuque, Iowa, USA) and left to imbibe the water for 1 h. Imbibed seeds were placed on 1% water agar (Sigma‐Aldrich, St. Louis, Missouri, USA) Petri plates overlaid with sterile germination paper (Anchor Paper Company, St. Paul, Minnesota, USA) and incubated at 26°C for 48–72 h. Seedlings with a radicle length of approximately 25 mm showing no signs of microbial contamination or necrosis were selected. Border cells were collected as described by Huskey et al. (2018).

### Preparation of RA staining solution

Five milligrams of RA disodium salt powder (Beantown Chemical, Hudson, New Hampshire, USA) were mixed thoroughly with 1 mL of sdH_2_O in a 2‐mL microfuge tube (some powder remains undissolved after shaking). One milliliter 0.2 M acetic acid was transferred to the mixture, shaken thoroughly, then centrifuged at 5000 rpm for 15 sec to pellet the undissolved powder and provide a particle‐free supernatant as the working solution in a clean centrifuge tube. Staining solution was prepared fresh prior to use.

**Figure 1:**
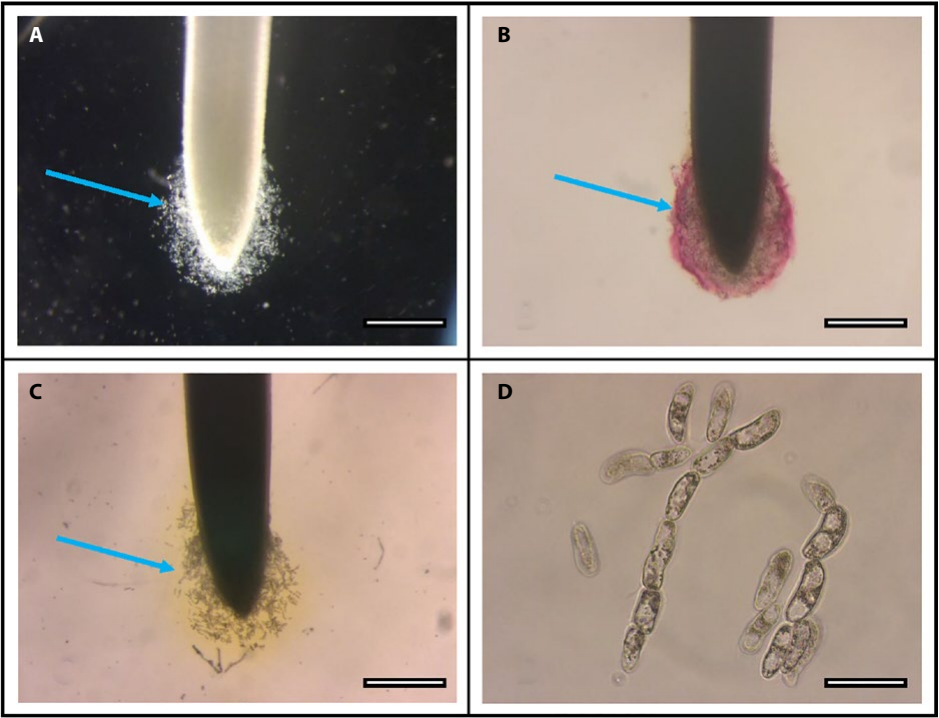
Use of rhodizonic acid (RA) to detect lead (Pb) trapping by border cells. (A) Border cells (blue arrows) surrounding a corn root tip immersed for 5 min in 1 mM Pb(OH)_2_. (B) The same root tip, 10 sec after dipping into RA staining solution for 10 sec, and transferring into sterile deionized water (sdH_2_O) for viewing. (C) Corn root tip immersed in Pb‐free sdH_2_O for 5 min, then stained for 10 sec with RA. (D) Border cells from (C). Scale bars: A–C = 1 mm, D = 50 μm.

Within 5 min of immersion of intact root tips into the Pb solution, expansion of border cells and mucilage surrounding root tips was evident (Fig. 1A) when visualized using a Zeiss SV8 stereo microscope (Carl Zeiss, Oberkochen, Germany). Images were captured with a Leica DFC290 HD digital camera (Leica Camera, Wetzlar, Germany) with Leica LAS software version 4.0.0 (Leica Microsystems, Heerbrugg, Switzerland). After exposure of the root tip to RA for 10 sec, trapping of Pb by border cells surrounding the same root tip was revealed by bright red staining characteristic of RA (Fig. 1B). When immersed into sdH_2_O and stained with RA in the absence of Pb, whole root tips (Fig. 1C) and their individual border cells (Fig. 1D) exhibit a pale yellow coloration in the surrounding mucilage. The RA stain is yellow when no Pb is present and does not exhibit the characteristic scarlet color.

**Figure 2:**
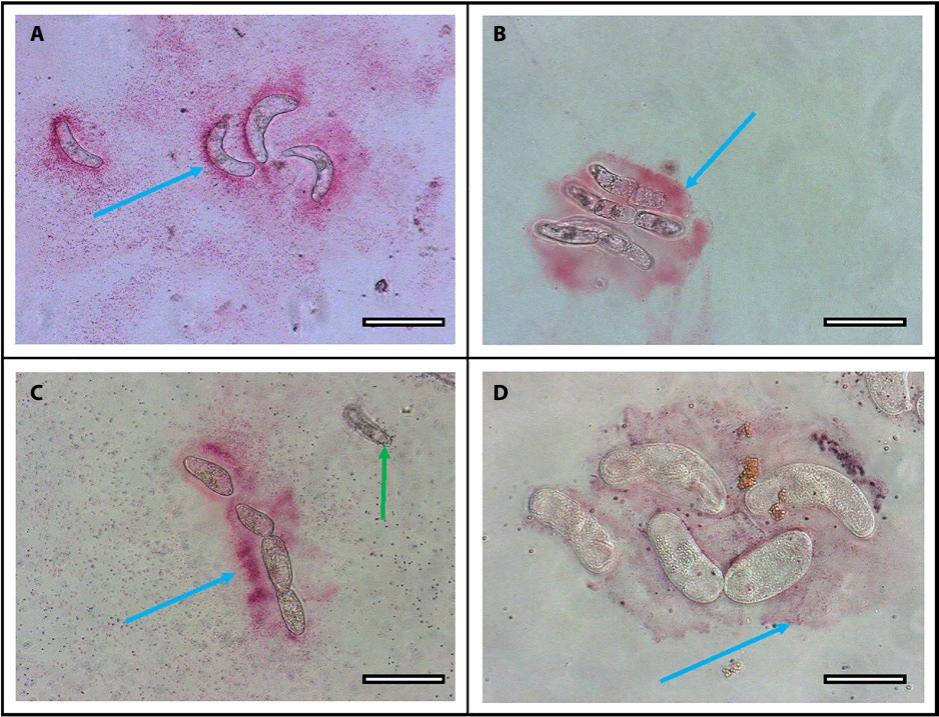
Corn border cell extracellular mucilage layer (blue arrows) in response to lead (Pb) solution. (A–C) Small clusters of border cells show bright staining of Pb at 200× magnification. Dead border cell (green arrow) did not produce a mucilage response and thus did not trap any Pb particles. (D) Diffuse mucilage staining and individual Pb particulates are evident at 400× magnification. Scale bars: A–C = 50 μm, D = 25 μm.

**Figure 3:**
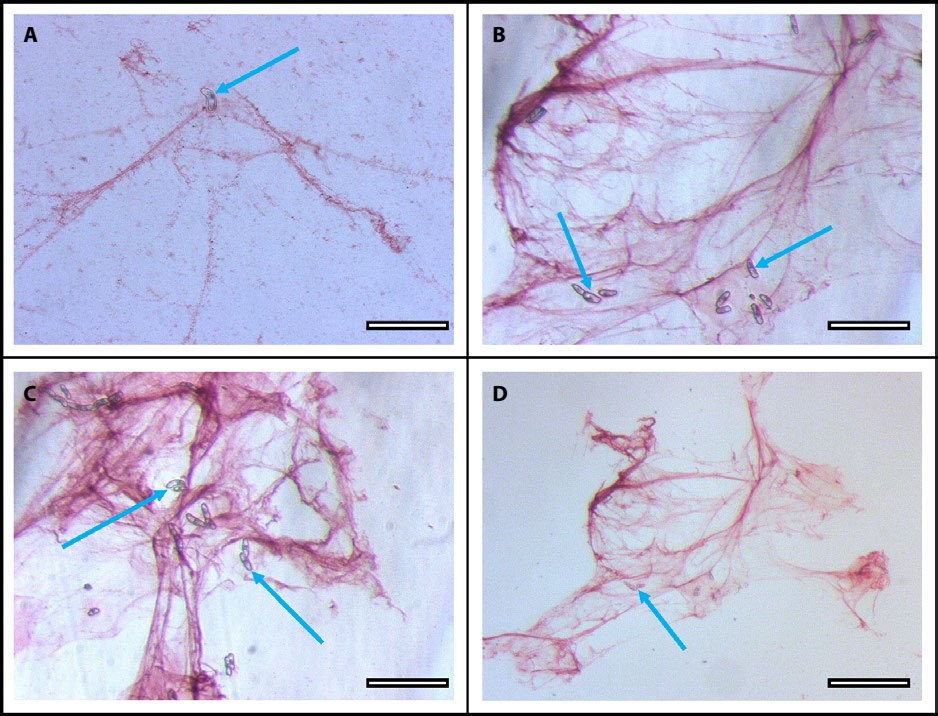
Diversity of corn border cell mucilage structure and scale in response to lead (Pb) stained with rhodizonic acid (RA). (A–D) Corn border cells (blue arrows) and mucilage incubated in 1 mM Pb(OH)_2_ for 1 h, then stained with RA. Scale bars: 200 μm. [Correction added on April 19, 2019, after first online publication: Figure 3 was replaced with an updated version.]

As previously reported (Huskey et al., 2018), the border cell capsule (mucilage layer surrounding individual border cells) expanded within minutes after exposure to Pb. After incubation of border cells with Pb for 60 min, accumulation of Pb particles around border cells (blue arrows) was revealed immediately following RA staining (Fig. 2A–D). Pb particles were trapped by the expanded mucilage in as little as 1 min, with increased trapping during longer exposure (data not shown). Trapping occurred only with living border cells; a single dead cell with collapsed internal structures and showing no cytoplasmic streaming (Fig. 2C) exhibited no RA staining. At higher magnification (Fig. 2D), individual Pb particles were evident within the traps. The magnitude of the trapping response over time was revealed by incubating a whole root tip in Pb(OH)_2_. After 60 min of incubation in Pb, border cells were gently transferred from the root tip to a microscope slide for visualization, without damaging the cell structure or viability (as in Curlango et al., 2013). Immediately after the application of RA, intensely stained diverse mucilage strands were revealed (Fig. 3A–D) surrounding the dispersed border cells (blue arrows).

### Treatment with nucleases

Two microliters of Ambion DNase I (2 U/μL; Thermo Fisher Scientific, Waltham, Massachusetts, USA), DNase II (30 U/μL; Worthington Biochemical Company, Lakewood, New Jersey, USA), or S1 nuclease (222 U/μL; Worthington Biochemical Company) were mixed with 50 μL 1 mM Pb(OH)_2_ (pH 5.5), and a root tip was immersed into the nuclease–Pb treatment solution. After 60 min of incubation, 25 μL of the suspension containing border cells and the nuclease–Pb treatment solution were gently removed and mixed with 10 μL of RA and visualized immediately with an Olympus BX60F5 compound microscope (Olympus Corporation, Tokyo, Japan). Efficacy of all three nuclease activities was confirmed by digestion of calf thymus DNA and gel electrophoresis with and without 1 mM Pb(OH)_2_ (data not shown).

**Figure 4:**
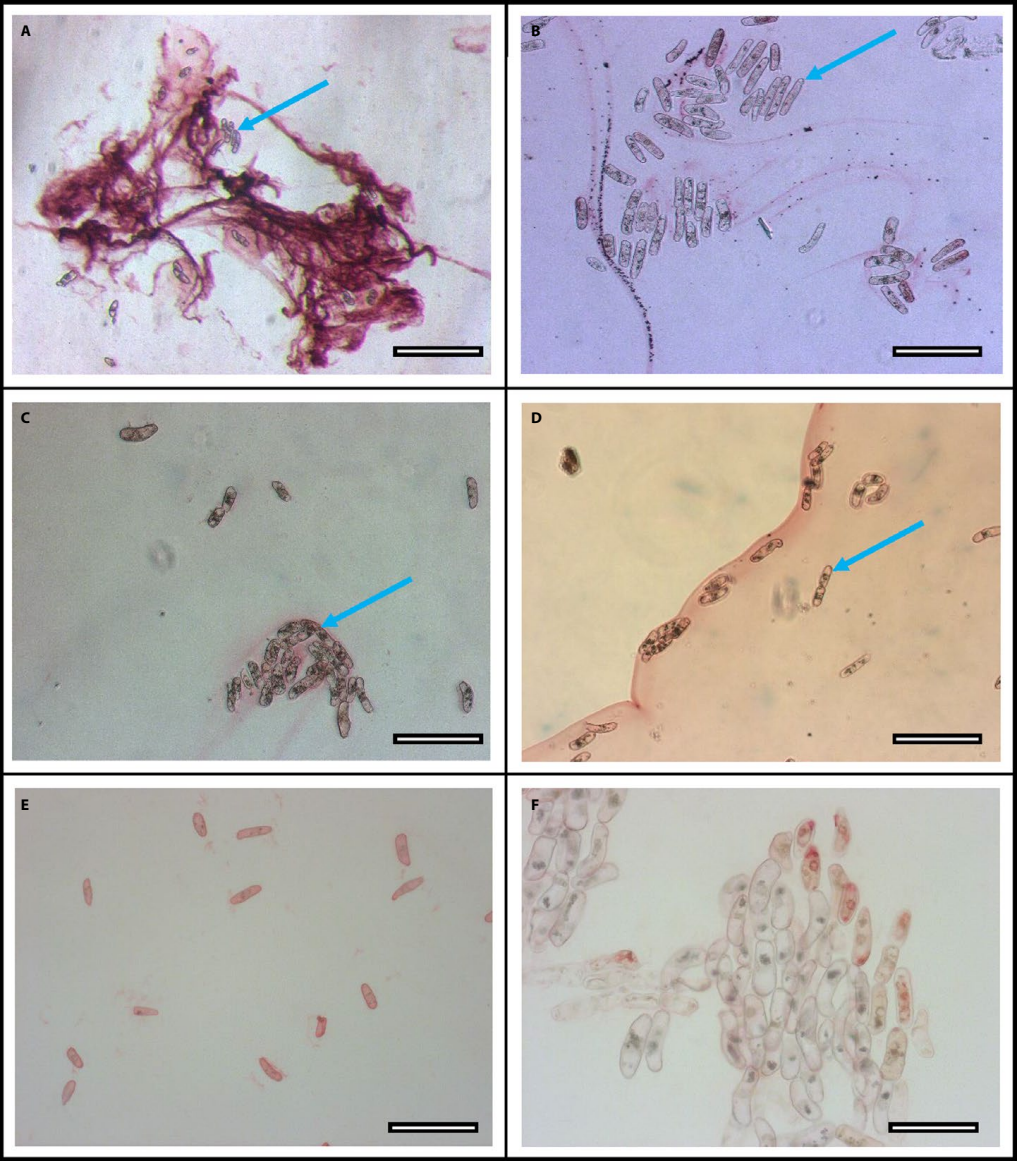
Inhibition of lead (Pb) trapping by corn border cells (blue arrows) in response to extracellular DNA degradation by nucleases. (A) Control corn border cells incubated with Pb and stained with rhodizonic acid (RA) after 1 h. (B) Corn border cells incubated with Pb and DNase II for 1 h and stained with RA. (C) Corn border cells incubated with Pb and DNase I for 1 h and stained with RA. (D) Corn border cells incubated with Pb and S1 nuclease for 1 h and stained with RA. (E, F) Corn border cells incubated with Pb for 1 h, then with DNase II for 20 min and stained with RA. Scale bars: A = 300 μm, B–E = 200 μm, F = 100 μm.

Mucilage structures that developed after 1 h in Pb followed by RA staining (Fig. 4A) were compared with mucilage samples incubated in Pb containing DNase II (Fig. 4B), DNase I (Fig. 4C), or S1 nuclease (Fig. 4D). Obvious dispersal of the mucilage traps occurred in response to all three nucleases. In addition, border cells incubated in Pb alone for 1 h, then treated with 2 μL of DNase II for 20 min and stained with RA revealed less Pb trapping than border cell samples incubated in Pb alone (Fig. 4E, F). Obvious dispersal of the mucilage traps occurred in response to all three nucleases, and the order of treatment application (Pb and nuclease simultaneously, or Pb followed by nuclease treatment) did not affect the reversal of trapping. This indicates that Pb trapping is reversible, even after adequate time for robust trap formation.

**Figure 5:**
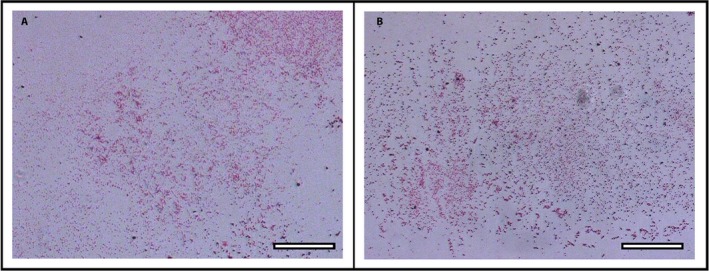
Effect of DNase on rhodizonic acid (RA) staining of lead (Pb) particles. (A) Pb particles were visible immediately after application of RA stain. (B) Pb particles remained visible after incubation with DNase II for 1 h and stained with RA. Scale bars: 100 μm.

These results suggest that exDNA is essential to the Pb trapping process. An alternative hypothesis is that the nucleases alter the chemistry of RA and prevent reaction with Pb. Direct tests did not support the premise that nucleases interfere with the ability of Pb to react with RA. When Pb alone was mixed with RA under the same conditions, red staining of Pb particles occurred throughout the suspension (Fig. 5A). No differences were observed when DNase II was included (Fig. 5B), and similar results occurred with DNase I and S1 nuclease (not shown).

## CONCLUSIONS

The use of plants to accumulate and remove heavy metals from contaminated sites by harvesting the aboveground parts is called phytoremediation (Pilon‐Smits, 2005; Kersten et al., 2017). Previous efforts to utilize plants in the uptake and removal of Pb have had limited success; among 450 plant species known as metal hyperaccumulators, Pb accumulators are rare (Fahr et al., 2013). Brassicaceae species, which are Pb accumulators, do not produce and deliver viable border cells (Niemira et al., 1996). Based on our results, it is reasonable to propose that border cells modulate uptake of Pb upon contact in the underground environment, and thereby prevent the effectiveness of plants as tools for Pb remediation (Clemens and Ma, 2016). Studies have revealed that Pb is abundant on the root surface and rhizosphere, but not the internal tissues, of rice and other plants (Lin et al., 2004). The magnitude and speed with which Pb is revealed by RA to associate with border cells is consistent with previous observations that border cell traps block uptake into the root tip (Huskey et al., 2018). This trapping mechanism could be used in the sequestration of toxic metals to stabilize soils and to prevent the leaching of contaminants into groundwater systems.

Our results suggest that trapping could be used as a new tool for phytoremediation by collecting roots ensheathed in trapped Pb, or by hydroponically passing contaminated water over roots. RA can potentially be used to define variables affecting Pb trap formation, quantity, and stability in response to soil type and other environmental variables. The surprising discovery that exDNA–DNase interactions are components of both mammalian and plant defense systems underscores the need for further research. DNA‐specific binding to metals has been studied in detail (Zhou et al., 2017), and understanding how exDNA–metal traps form and function could yield key insights into plant, animal, and soil health. As previously established, nuclease degradation of exDNA in border cell traps results in hampered trapping capacity of both abiotic and biotic stressors (Hawes et al., 2016a; Tran et al., 2016; Wen et al., 2017; Park et al., 2019). There is potential to improve agricultural pest management and remediation of contaminated sites through exploitation of this exDNA‐based mechanism, perhaps by identifying specific DNA sequences that preferentially bind to metals and microbes. The use of RA for real‐time detection of Pb in, on, and around living plant tissue is a tool that merits further investigation.
